# Structural and relational factors for successful cross-sector collaboration in home visiting: a multiple case study

**DOI:** 10.1186/s12913-024-10719-4

**Published:** 2024-03-08

**Authors:** Venice Ng Williams, Carol Yvette Franco-Rowe, Connie Cignetti Lopez, Mandy A. Allison, Gregory J. Tung

**Affiliations:** 1 Prevention Research Center for Family & Child Health, University of Colorado Anschutz Medical Campus, 1890 N. Revere Ct, MS F443, 80045 Aurora, CO USA; 2 Colorado School of Public Health, 13001 E. 17th Place, MS B119, 80045 Aurora, CO USA

**Keywords:** Cross-sector collaboration, Care coordination, Integration, Home visiting, Family well-being, Maternal child health, Case study

## Abstract

**Background:**

Aligning delivery and financing systems across sectors to create broader systems of care can improve the health and well-being of families experiencing adversities. We aimed to identify structural and relational factors for best practices to achieve successful cross-sector collaboration among home visiting programs in the United States.

**Materials and methods:**

We used a multiple case study approach to identify best practices for successful cross-sector collaboration between home visitors and other community service providers. We selected five diverse exemplary cases with cross-sector collaboration with variation in implementing agency type and geographic location. Cases were selected using a positive deviance approach based on strong coordination and integration with different community service provider types identified from previous survey data. We conducted in-depth qualitative interviews with home visiting staff, community providers, and clients with a total of 76 interviews conducted from 2021 to 2022. We wrote memos to synthesize themes within each case through data triangulation using interview data, documents, and site visit observations. We compared themes across the five cases to create a cross-case synthesis of best practices for successful cross-sector collaboration.

**Results:**

Across the five cases, relational factors including leadership from all levels, champions across sectors, and shared goals between community providers were key factors for successful collaboration. Interpersonal relationships, coupled with the desire and capacity to engage, facilitated effective coordination to address families’ needs. At the structural level, shared data systems, written agreements, and co-location enabled care coordination activities. Community Advisory Boards provided a venue for developing partnerships, relationship-building, resource-sharing, and increasing awareness of home visiting.

**Conclusions:**

We identified key elements of successful cross-sector collaboration across five case studies where home visitors coordinate care frequently and/or are structurally integrated with a range of providers. These learnings will inform future interventions to improve home visiting collaboration with other community providers to create a system of care to enhance family well-being.

**Supplementary Information:**

The online version contains supplementary material available at 10.1186/s12913-024-10719-4.

## Introduction

Child poverty is greater in the United States (US) than in most other countries with similar resources [1]. More than one in three children in the US live in families with incomes at or below 200% of the federal poverty threshold, [2] which places children at greater risk [3] for adverse childhood experiences that often lead to lifelong negative effects on health [4]. Public health programs use home visiting to help families achieve economic self-sufficiency and prevent adverse outcomes associated with poverty. Home visiting refers to programs where a professional or paraprofessional provides a service in the community or private home setting [5]; or where home visitors are a central component of a broader service plan [6]. These programs range in intended population, program goals, and evidence for improving maternal child health outcomes [5, 7]. Home visiting programs are part of the broader systems of care to improve family well-being.

Families experiencing adversity often interact with multiple systems to meet basic needs [8]. Home visiting is well integrated into the broader system of care for families in the international setting [9, 10]. In the US, however, systems offering such programs are not designed to function cohesively; they communicate infrequently or ineffectively, with little alignment in purposes [11, 12]. Aligning the delivery and financing systems for medical care, public health and prevention, and social and community services to create broader systems of care can improve the health and well-being for all people [13], particularly young children and their families living in poverty [14]. The system of care approach combines a range of services and supports with guiding principles and core values to meet the needs of each child [15]. Systems of care have been found to be associated with improvements in the lives of families through improved child wellbeing, child safety, and family functioning and improvements in service delivery such as increased use of evidence-based practices, improvements in care management, and better investment of resources [16, 17].

Strengthening home visitors’ collaborative practices with community service providers across sectors [18] may support building systems of care to more efficiently and effectively address issues of childhood poverty. Home visitors’ ability to address maternal and child health risks can be improved through better integrating approaches and systems across public health, medical care, and social services. This integration of services and systems would ensure that families receive needed services and continuity of care [5] through implementation of wraparound approaches to service planning and delivery with family involvement and care coordination [19].

Nurse-Family Partnership (NFP) is a home visiting program with strong evidence for improving health and well-being of first-time pregnant and parenting people and their children experiencing economic, social and/or physical health adversities. The program is based on over 40 years of evidence from three separate randomized clinical trials in distinct communities [20–22]. The program began community replication in the US in 1996 and has now served over 385,000 families [23]. NFP aims to improve pregnancy outcomes, improve child health and development, and increase families’ economic self-sufficiency. Eligible participants receive visits from trained nurses early in their pregnancy through child age two, receiving support, education and linkages to needed community services. Nurses follow program protocols that are grounded in theories of developmental epidemiology, human attachment, human ecology, and self-efficacy and are adapted to meet families’ individual needs [24].

Previous studies have shown that key factors for collaboration in the early childhood setting include the role of program champions, sustainable structures, interagency agreements, common performance metrics and goals, relationships and leadership support, and alignment with other state or community efforts [25, 26]. Most previous investigation of cross-sector collaboration specific to home visiting has been limited to screening and referrals [27–29]. One recent multiple case study explored service coordination in early childhood home visiting and found that relationships at multiple levels are important, barriers are complex, and coordination is time-intensive [30]. Our previous research about collaboration in NFP has mainly focused on single provider types in specific geographic regions. We found that the alignment of organizational mission and approach to family engagement in service delivery supported stronger collaboration between child welfare and nurse home visitors (NHV) in Colorado [31]. A single case study in the Northwestern US identified how and why NHVs coordinate care with healthcare providers [32]. Another study identified factors that contributed to effective collaboration between NFP NHVs, healthcare, and community support services, including relational (leadership commitment and provider champions), organizational (mission congruence), and structural (policy and systems integration) elements [33].

Despite previous research in this area, there remains a strong need to understand the common factors that lead to the effective implementation of systems of care between public health home visiting nurses, medical providers (including women’s care, pediatric care, behavioral health), and social service providers (like child welfare, supplemental nutrition, housing, early intervention, etc.). Therefore, we aimed to identify structural and relational factors for best practices to achieve successful cross-sector collaboration to build effective systems of care for families engaged in home visiting. We implemented a positive deviance approach using survey data to identify highly collaborative NFP programs in the US to conduct five individual case studies. Findings across the five cases informed this multiple case study.

## Methods

### Study design

We used a multiple case study approach to identify best practices for successful cross-sector collaboration and building effective systems of care. We adopted Yin’s definition of case-study research, [34] and gathered perspectives from multiple roles (NFP NHVs, community providers, NFP clients) to triangulate and reconcile differences to understand collaborative dynamics within each community context. The cases were bound by geography (within the counties served by NFP NHVs). The interviews with NFP NHVs and community service providers were approved by the researchers’ local Institutional Review Board as exempt research, and client interviews were approved as human subjects research. No incentives were provided to NFP NHVs and community service providers given their participation in interviews were only related to their professional experience. Clients who completed an interview received a $40 gift card.

### Operational definitions

**Fig. 1:**
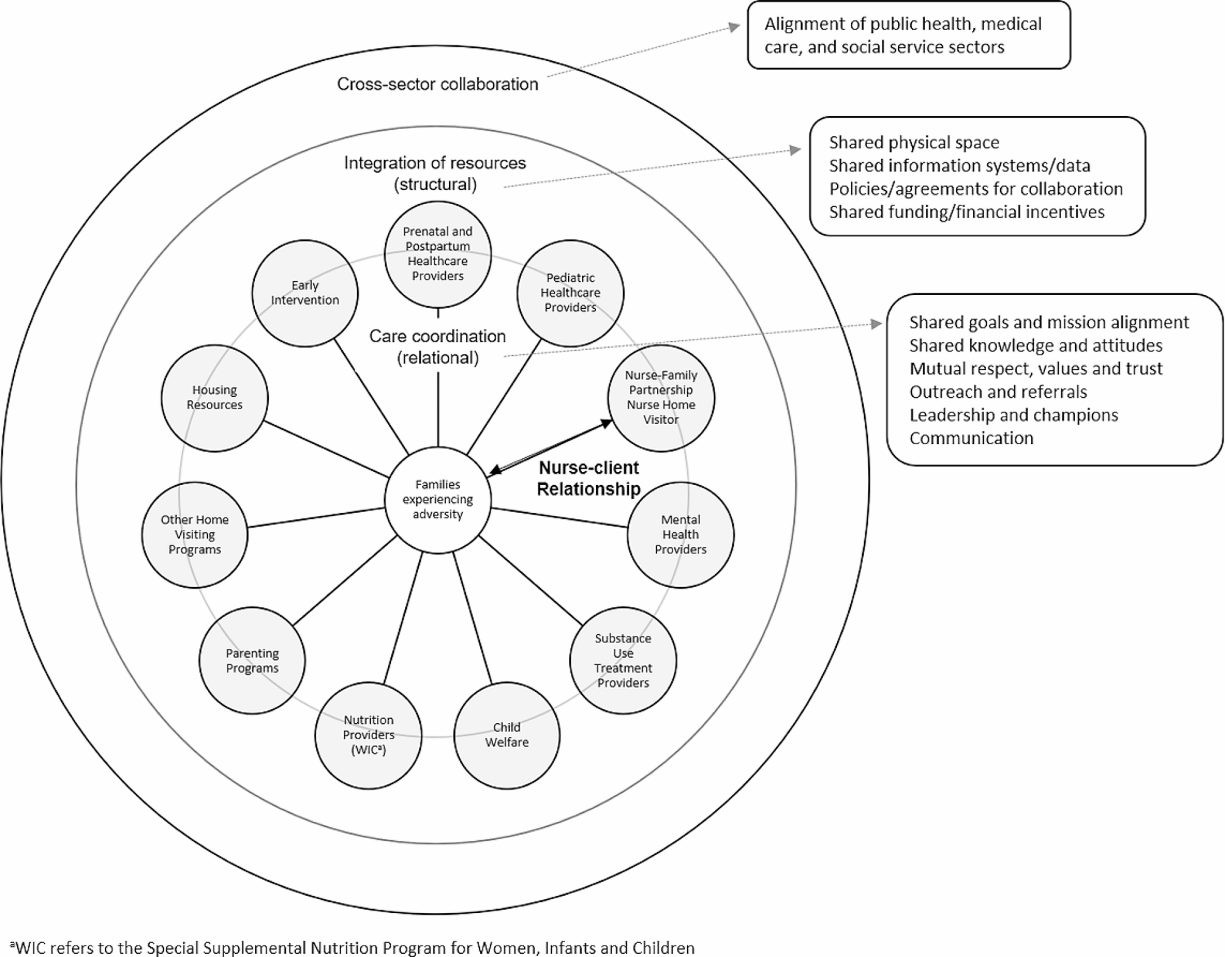
Theoretical Framework of Collaboration.

We define *cross-sector collaboration* as the alignment of service delivery and/or financing systems across sectors of public health, medical care, and social services [35]. Collaboration involves multiple systems, organizations, and service providers collectively focusing their expertise and resources on addressing complex community issues through shared goals. Care coordination and integration of resources are essential components of collaboration. We define *care coordination* as the deliberate organization of activities between two or more providers to facilitate, in partnership with the family, the appropriate delivery of needed services [36]. *Integration of resources* refers to the extent that infrastructures are shared between organizations, such as information systems, protocols or agreements, space, and accountability or rewards [37]. Coordination is driven by *relational* dynamics between people, while integration involves the *structural*nature of organizations. Figure 1 depicts our conceptual understanding of these terms and their interrelatedness, drawn from our prior research [33, 38] and influenced by relational coordination theory [37].

Primary data for the case studies were collected from 2021 to 2022 with five exemplary NFP programs that collaborate with community providers in health care and social services. We used a multi-step process to identify potential cases for inclusion informed by a positive deviance approach, which involves identifying and learning from those who “demonstrate exceptional performance on an outcome of interest” [39]. First, we used survey data collected from the 2020 NFP Collaboration with Community Providers survey that measured coordination and integration with providers in health care and social services from the perspective of NFP nurse supervisors. Coordination was measured by high-quality relationships (shared goals, shared knowledge, mutual respect) coupled with high-quality communication (frequent, timely, accurate, problem-solving communication) [37]. Integration was measured by the degree of sharing of resources including physical space, data or information systems, policies or agreements, and funding [38]. The survey is described in more detail elsewhere [38, 40]. We then identified programs that scored in the top fifth percentile in their reported scores of coordination with substance use treatment providers, child welfare, and/or nutrition and/or integration with women’s care, pediatric care, and/or child welfare. Collaboration with these specific provider types were selected due to their strong correlation with improved client and nurse retention in the NFP program. Next, we populated the programs’ rates of client retention and only included those with a higher retention rate than the national NFP program average. Finally, we used a maximal variation approach to select programs that varied by key contextual characteristics, such as program size, geographic location, rurality of community served, and type of agency implementing the program [41]. We approached seven programs and five agreed to participate: an urban health system in South Central US, a rural health department in Western US, a statewide community-based organization in Northeastern US, an urban health department in Southeastern US, and an urban health system in the Mid-Atlantic US.

### Data sources

We conducted case studies in partnership with each NFP program. Three researchers with backgrounds in anthropology, public health, and nursing collected and analyzed the data for the five cases. One researcher led each case study with support from the others as needed to collect and analyze data. For each case, we conducted in-depth interviews first with NFP staff, who then identified community service providers with whom they collaborate, current NFP clients who receive care coordination from their NFP nurse, and NFP graduates (past participants) who had experienced care coordination during their participation in the program.

### Data collection

Between May 2021 and April 2022, three researchers conducted interviews with a total of 79 participants across the five cases (see Appendix A); with 51.5% of participants representing NFP (*n* = 37), 28.5% representing collaborative partners (*n* = 22), and 20% representing client perspectives (*n* = 20). Among the collaborative partners, 27% worked in public health (*n* = 6), 41% worked in medical care (*n* = 9), and 32% worked in social services (*n* = 7). Interviews lasted 30 to 90 min and were recorded with the participant’s verbal informed consent. We used a thematic interview guide with sample probes (see Appendix B for full guide). We began by asking about the participant’s professional role/experience and their organization’s structure as context. Then, we moved to explore topics of coordination and integration with the relevant provider type as identified from the survey data. For example, the NFP program in Case 1 reported strong coordination and integration with women’s care providers in the 2020 survey, so the interviewer explored these topics with participants. To characterize coordination, we inquired about topics of outreach, referrals, knowledge and attitudes, values and trust, mission alignment and shared goals, leadership and champions, and examples of care coordination. To characterize integration, we explored topics of shared physical space, shared information, policies for collaboration, and financial incentives. We did not ask the topical themes in a particular order but directed the conversation with probes to allow participants to weave their own narratives while ensuring that all topics were covered [42, 43]. Because one NFP program was located in the same state as the research team, we conducted a site visit in one case, touring and observing at the site, and attended a virtual Community Advisory Board (CAB) meeting. CABs are a required element of the NFP model and intended to support program implementation and sustainability.

### Data analysis

After each completed interview, the interviewer listened to the recording and wrote an analytic memo to abstract emerging themes discussed during that interview. When appropriate and available, documents such as written policies and site visit observations were used as an additional data source for the cases. We used a data triangulation and explanation building approach to synthesize the data from the interview memos and documents [34]. This approach involved using multiple data sources (e.g. multiple interviewee perspectives) to explain and explore the phenomenon of interest and iteratively developing an explanation of key factors for collaboration and their linkages for each case [34]. The lead researcher then compiled thematic memos by examining each theme across interview memos to create each case study. To promote credibility, thematic memos and the full written case study for each case were shared with other team members and an Advisory Board with experience in prevention science, health services research, pediatrics, biostatistics, nursing, child welfare, and the NFP program for feedback and expert validation. The first author then compared themes across the five case studies to conduct a cross-case synthesis to identify common best practices for collaboration.

### Findings

We provide a summary of each case below followed by the cross-case synthesis. See Appendix C for additional details for each case.

#### Case 1: rural health department in Western US

This program was selected for reported strong integration and coordination with women’s care providers and is implemented by the local health department (LHD). The LHD serves a rural and agricultural community with an urban center, and the NFP client population is primarily White, Hispanic/Latin American descent or origin, and of refugee status. A regional health alliance, which includes the LHD, federally qualified health centers, and a behavioral health agency, share an electronic medical record (EMR) system, which facilitates communication between providers. The NFP program is also co-located with the family planning clinic located in the LHD where nurse home visitors (NHV) can attend appointments with clients.

NHVs primarily coordinate with women’s care providers from the three entities in the regional health alliance, which centers around a shared mission, mutual awareness, program knowledge, leadership support for coordination, and program champions for referrals. This program has strong coordination with other programs including the LHD social worker for client resources, Supplemental Nutrition Program for Women, Infants, and Children (WIC) for clients’ nutritional needs and breastfeeding supports, United Way for client items (like diapers or car seats), the local library, and a local university reading program. Members from these organizations participate in this program’s CAB. Virtual monthly CAB meetings, chaired by the nurse supervisor, facilitate ongoing communication between the programs regarding resources and events, while promoting strong member relationships.

#### Case 2: urban health system in South Central US

This program was selected for reported strong integration with women’s care and pediatric care providers and is implemented by a teaching health system under the women’s services line. The health system has 12 satellite ambulatory clinics (10 offer women’s care and pediatric services where NFP clients and their children receive medical care) and has a medical insurance navigator to enroll patients, and an obstetrics patient navigator who welcomes patients into the system. Based in a primarily urban-suburban community, approximately 20% of NFP clients are monolingual Spanish-speaking, while 10–15% are undocumented or refugees. NFP and the health system share a medical campus where NHVs have badge access to clinics and the hospital. NHVs have read-only access to the shared EMR to view clinic notes and send messages and the nurse supervisor has full access to the EMR. Access to clinic notes requires signed consent forms and releases of information by NFP clients.

Shared goals of compassion, collaboration, and education for patients as well as shared outcomes of healthy pregnancies and deliveries are hallmark to NFP and the women’s health services line. However, the approach to delivering care differs between NFP and women’s health providers. Patient navigators and clinic staff serve as referral partners and program champions of NFP. Personal relationships at the frontline and supervisory levels facilitate regular communications. The CAB for this NFP program is shared with two other NFP programs located in the same community, which maximizes member attendance, allows for efficient sharing of performance metrics and community experts, and reduces duplication of meetings.

#### Case 3: urban health department in Southeastern US

This program was selected for their reported strong coordination with WIC and child welfare and integration with women’s care providers and is implemented by the LHD under the Maternal Child Health Division, alongside four other home visiting programs, a family counseling service, and WIC. The LHD has six health centers that offer pregnancy confirmation services, one of which offers obstetric care service. The community consists of urban and suburban areas, where the NFP population is comprised of White, Black, and Hispanic/Latin-X. The LHD has a shared EMR system that allows providers within the department to view visit information after releases of information are signed. NHVs also have badge access to the health centers. Care coordination and program referrals are facilitated by strong personal relationships between NHVs and women’s care providers.

NFP and child welfare value safe pregnancies, child health, and safety, with common goals of primary (preventing families from entering the system) and tertiary prevention (connecting entering families with wrap-around services). This program has additional relationships with a related but separate entity focused on child maltreatment prevention, which provides NFP funding, supports NFP implementation and consultation, and champions the program in the community.

NHVs primarily coordinate with WIC through referrals, where clients enroll on their own as they are empowered to self-advocate using WIC’s streamlined enrollment process. Respect for WIC’s breastfeeding and nutritional expertise and shared goals to support healthy mothers and babies facilitate this process. Despite co-location, NFP rarely coordinates with WIC beyond referrals.

#### Case 4: Community-based organization in Northeastern US

This program was selected for reported high levels of coordination with substance use treatment and child welfare services and is implemented by a non-profit community-based organization (CBO) housing 40 programs through multiple sites in and around the city center. The NFP program serves all families within the state, mostly in urban and suburban areas, who are primarily White, Black, Hispanic/Latin-X, and those with undocumented or refugee status. The small size of the state allows it to function like a community where community leaders and service providers share commitment to improve family well-being.

Coordination with substance use treatment exists with a specific home visitation and services referral program for expectant parents (the “Project”) that are affected by substance use and involved with child welfare The Project is housed in the same CBO as NFP and falls under the jurisdiction of the state health department because of their funding stream, like NFP. Coordination revolves around sharing of information, conducting joint visits, and case conferences to discuss client needs.

Mission alignment exists between NFP and state child welfare, with the primary goal of family reunification and preservation, knowing that these families need reliable supports in place to help them succeed. State child welfare perceives NFP as a reliable program for families involved in child welfare. Releases of information allow for sharing of information and NHVs coordinate with caseworkers to discuss client’s service plans.

#### Case 5: urban health system in Mid-atlantic US

This program was selected for reported strong coordination with substance use providers and integration with women’s care and pediatric care providers and is implemented by a teaching health system under the women’s and pediatric services line along with obstetrics and gynecology, pediatrics, the Centering program, family planning, a home visitation program for pregnant persons eligible for medical assistance (Medicaid), and a substance use program for mothers affected by substance use. The NFP team serves clients who are White, Black, Hispanic/Latin-X, and refugee populations living in urban areas, suburban areas, and rural/agricultural surrounding areas.

The health system has a shared EMR system that allows all providers within the department to see if a visit was made. NHVs can chart, view clinic notes, send messages to providers, and send referrals. Within the health system, all pregnant people are screened for eligibility for home visiting services at their first prenatal appointment. The Medicaid home visitation program at the health system receives these referrals and outreaches to the referrals, conducts a full assessment including mental health needs, and then refers them to the appropriate service.

Coordination with substance use relates to clients dually enrolled in NFP and the health system’s substance use home visitation program. The two programs share an administrator, the same office space, and EMR system, which facilitates communication and coordination.

### Cross-case synthesis

We identified relational and structural factors that led to successful cross-sector collaboration across all cases. Collaboration was successful when NFP clients received referrals and were utilizing the resources they needed and communication between NHVs and community service providers was efficient and occurring as needed. Relationally, leadership from all levels, champions across sectors, and shared goals between community providers to support families experiencing adversities were key factors for successful collaboration. Structurally, shared data systems, written agreements, and/or co-location typically enabled care coordination activities. See Table 1 for a comparison of similarities and differences between cases.

### Relational factors

In all cases, leadership support of the NFP program was identified from the executive level to managerial and front-line service delivery staff, which motivated providers from cross-sectors to collaborate with NHVs when working with mutual clients. In Case 1, a well-respected community leader initiated the NFP program, while in Case 3, leadership from the local funding agency identified NFP as the program with the strongest evidence that would be able to address the child maltreatment rates in their county which were the highest in the state. In Cases 3 and 5, the NFP administrator also oversees other programs serving similar populations and encourages collaboration between the various services. In Case 4, leadership from two state agencies promote provider collaboration and communication across sectors and programs.

Specific individuals were often named as champions of NFP. These champions mostly worked in healthcare (typically nurse managers, social workers, patient navigators, or intake coordinators), the nonprofit sector (in child maltreatment prevention), public health (like WIC) and social services (such as other parenting or early childhood programs). Many providers with a background in public health or social work were NFP champions. Champions of NFP were most helpful in referring eligible clients to the program, connecting NHVs to specific providers for coordination purposes, and supporting clients in reaching their goals.

Shared goals between NHVs and community providers often involved supporting families experiencing the greatest psychosocial or socio-economic adversities. Across all cases, participants identified organizations and providers with whom they collaborate and the importance of sharing a mission, goal, and/or approach to working with families. A commonly shared goal in all cases was to promote healthy pregnancies, births, and babies. Community service providers who used a strength-based, family-centered approach were more likely to effectively coordinate with NHVs.

Interpersonal relationships between NHVs and community service providers, and the desire and capacity to engage, further facilitated effective coordination to address families’ needs. The desire and capacity to engage related to whether or not leadership supported and prioritized collaboration. Providers who coordinated effectively with NHVs tended to understand the value of NFP and had the time and/or tools to communicate as needed. In all cases, the NFP nurse supervisor and several NHVs had personal relationships with providers working in healthcare and community-based organizations from previous work history or from living in the same town. In Case 1, NFP is implemented in a rural community where NHVs and community service providers interact outside of the professional setting. In Case 4, NFP serves a small state that functions like a “small town” where providers rely on one another to collectively support the well-being of families.

### Structural factors

Shared data systems, usually EMRs, were present in four of five cases. Two of these cases were NFP programs implemented by health systems and two were implemented by LHDs. The only case without shared data systems with health providers was the NFP program implemented by a CBO; however, they have an internal EMR system where in-house programs can see which clients are enrolled and their demographic information which facilitates collaboration with dually-enrolled clients. While the degree of shared systems ranged from view only to full access, they offered processes for effective collaboration. Shared data systems in all cases allowed for efficient referrals, both to NFP and other services like healthcare, WIC or other home visiting; communication channels through messaging and “flags”; identification of previous or active participation in various services like child welfare; and review of adherence to appointments and any notes of concern, such as those that are health-related.

Written policies and agreements were another structural factor that facilitated effective communication and coordination. When NFP is implemented by an organization that offers other programs and services, they usually share the same policies and protocols, i.e. informed consent with clients. This sharing of policies occurred in all cases with different providers, like women’s and pediatric care, substance use treatment, WIC, and other parenting programs. In all cases, formal Memoranda of Understanding (MOU) or releases of information were in place to allow NFP to share information regarding their mutual clients and collaborate with providers outside of the implementing agency.

Co-location or sharing of physical space was present in four of five cases. Co-location did not always lead to strong coordination and communication and varied by provider type. Most often sharing of physical space, like a campus or building, allowed for NHVs to more efficiently enroll clients into the program or to coordinate services with other providers. For example, in Cases 1, 2, and 5, NHVs often attended health visits with their clients as they had badge-access to the clinic or hospital. While in Case 3, co-location with nutritional providers did not promote strong coordination.

### The role of community advisory boards (CABs)

NFP programs are required by the NFP National Service Office to have a dedicated CAB that supports their implementation. CABs from each case ranged in size, goals, and effectiveness. The more effective CABs provided a venue for developing partnerships with providers across sectors, relationship-building, resource-sharing, and increasing awareness of NFP. Case 1 had the strongest CAB where organizations serving the same population actively engaged and participated in meetings. They had a shared desire to ensure that families received the care they needed with the resources available in the community, which was possible through creation of an informal system of care to meet the unique needs of each family engaged in multiple services.

## Discussion

We aimed to identify common factors that contribute to effective collaboration between home visitors and other community service providers across sectors to create systems of care that improve family well-being. This study adds to our previous survey work that quantified measures of collaboration in the home visiting setting from the perspective of NFP nurse supervisors as a representative of their site. This study uses a qualitative approach, which allows us to understand nuances and triangulate the perspectives and experiences of NHVs, NFP families, and community service providers from five exemplary cases on a complex, nuanced dynamic. Each case presented unique collaboration dynamics with providers in the fields of healthcare, public health, and social services. However, we identified key elements of successful collaboration across all cases, including relational factors: leadership, champions, shared goals, and the importance of relationship maintenance; and structural factors: integrated systems through shared data systems, policies and agreements, and physical space.

Previous research has identified the importance of collaborative champions who serve as formal systems leaders, [44, 45] where the role of the champion varies by sector [32]. Champions motivate front-line staff to collaborate; where motivation and capacity to integrate purposes and action across partners is necessary for successful collaborative activities [46, 47]. Mutual respect and understanding among partner organizations [43] and having a shared vision, language, and common goals [33, 45, 48–50] also facilitate coordination. Our study supports these findings and adds that personal relationships play a role in ongoing and effective communication. These strong relationships are often due to providers’ value of the NFP program, their professional background aligning with the program (i.e. public health or social work experience), and their positive experience of the program (e.g. NFP clients were more likely to attend health visits or make progress on their goals). To achieve this in practice, home visitors may consider ongoing relationship maintenance activities like personalized outreach, quarterly e-communications about their program or physically visiting their workplace annually to drop off program brochures once they identify a specific service provider who knows and understands the program. These outreach activities require protected paid time.

Previous research has identified the role of integrated information and communication infrastructures like shared electronic record systems [33, 44] and shared data and measurement [50, 51] in supporting cross-sector collaboration. Co-location or sharing of physical space provide organizational opportunities for shared trainings, administrative efficiencies, [52] and regulatory policies and mandates [33, 44] enable information sharing and communication between providers. Our study and others show that co-location aids but does not necessarily improve collaboration [53]. In addition to these factors, our study offers specific examples of how the use of informed consent forms, releases of information, and Memoranda of Understanding (MOU) vary based on the type of agency implementing NFP and provider. For example, programs housed within the same organization typically only need signed releases of information, while external partners execute a formal MOU. We also learned that execution of these agreements with external agencies requires relationship building and program champions to articulate the need for sharing personal health information to address families’ needs. This can include sharing of sample scenarios where access to and knowledge of a family’s medical and/or social history was essential to co-creating a shared care plan.

While previous research has described the participation of community service providers on NFP CABs [33], our study provides specific examples of how a strong CAB motivates its members to support the implementation of NFP and ensures that families receive needed care and services. Strong CABs provide opportunities for ongoing relationship maintenance so providers and NHVs see one another in the community, share resources, and problem solve together. To support ongoing relationship maintenance, interprofessional education [44] and participation in community coalitions creates program exposure to remind providers of the NFP program [33]. However, these factors were not discussed by participants in any of the five cases as facilitators to collaboration. This may be due to the case selection process where we used a positive deviance approach. These factors may be more necessary for building relationships, rather than maintaining them, and the cases we selected had already developed strong provider relationships.

### Strengths and limitations

Our study limitations include the specific focus on NFP, data collection from highly collaborative NFP programs identified through self-reported survey data, and exploration of collaboration with women’s care, pediatric care, substance use treatment, child welfare, and nutrition which excludes providers from other sectors that intersect with home visiting. We also recognize that these findings may not be generalizable to all home visiting programs, particularly those that employ para-professionals who may be more limited in their abilities to communicate with other healthcare providers. We acknowledge that prior research on collaboration utilize similar terms surrounding “collaboration” as in our study and may have the same or slightly varied meaning, a different meaning or no formal definition. Our discussion of this study’s findings integrates prior research based on our assessment of their results regardless of terminology used. The usefulness of research is constrained when there is misalignment in terminology. As such, improving the clarity and precision of our terminology is needed to advance learning more effectively.

Despite these limitations, our study provides nuanced learnings unique to the community context of each case and identifies findings that are consistent across settings. These consistent learnings are likely transferable to similar settings in the US that implement community-based programs using a strengths-based, family-centered approach. Our triangulation of multiple perspectives and data sources and cross-case examination allowed for a comprehensive and holistic exploration and explanation of cross-sector collaboration in the home visiting and prevention program context.

### Implications for practice, research, policy

Home health visiting is a valued, effective service with a long history of implementation in the international setting. Countries that offer these services include Denmark, [54] Australia, [55, 56] Canada, [57] and the United Kingdom, [58] which all have comprehensive maternal child health systems where home visiting is well integrated into the broader system of care for families. This model of integration is rare in the US which creates an opportunity to address childhood poverty and other social determinants of health through greater systems alignment across sectors. The best practices identified in this study, including relational and structural factors for effective cross-sector collaboration, can inform practice tool-kits and future interventions to improve home visiting collaboration with other community providers to create a system of care to enhance family well-being.

**Table 1:** Comparison of Five Cases

	Case 1	Case 2	Case 3	Case 4	Case 5
Implementing agency type	Local health department	Teaching health system	Local health department	Community-based organization	Teaching health system
Location and area(s) served	Western United States, rural/agricultural surrounding areas	South Central United States, urban/sub-urban	Southeastern United States, urban/sub-urban	Northeastern United States, statewide, urban/sub-urban	Mid-Atlantic United States, urban/sub-urban/rural-agricultural surrounding areas
Reason for selection	Strong coordination and integration with women’s care	Strong integration with women’s and pediatric care	Strong coordination with WIC and child welfare; Integration with women’s care	Strong coordination with substance use treatment and child welfare	Strong coordination with substance use treatment; Integration with women’s and pediatric care
Year began NFP implementation	2001	2008	2012	2009	2001
Funded capacity and source(s)	150 clients from state funding	200 clients from federal Medicaid block grant	120 clients from local non-profit children’s council	200 clients from the federal Maternal, Infant, and Early Childhood Home Visiting program	290 clients from blended state funding
Organizational and team structure	Under clinical and community health division with 1 nurse supervisor, 1 administrator, 6 nurse home visitors (NHV)	Under the women’s service line with 1 nurse supervisor, 1 administrator, 8 NHVs, and 1 data entry clerk	Under maternal child health division with 1 nurse supervisor, 1 administrator, 6 NHVs, 1 records technician, 1 contracted mental health counselor	Under home visitation division with 1 nurse supervisor, 1 nurse manager, 6 NHVs (2 bilingual)	Under the women’s and pediatric service line with 2 NFP teams: 2 nurse supervisors, 1 administrator, 12 NHVs, 1 administrative assistant
Relational facilitators	Leadership support Community buy-in Program champions and reliable referrals Shared mission, goals and approach to care Interpersonal relationships Strong nurse supervisor Shared client population	Leadership support Program champions and reliable referrals Shared mission and goals Shared client population	Leadership support Shared mission and goals	Leadership support Community buy-in Shared mission, goals, and approach to care Shared client population	Leadership support Program champions and reliable referrals Shared mission and goals Interpersonal relationships Common administrator for multiple programs Shared client population
Structural facilitators	Co-location and close proximity Written agreements Full access to shared electronic medical records (EMR) system	Badge access Common internal policies View-only access to shared EMR (full-access for nurse supervisor)	Co-location Written agreements Full access to shared EMR system Access to state data systems (child welfare) Funding sustainability	Co-location and close proximity Common internal policies Internal EMR system Centralization of programs/services State oversight	Co-location and badge access Common internal policies Full access to shared EMR
Community Advisory Board (CAB) functioning	Strong engagement from diverse sectors Meetings facilitate ongoing communication	Statewide CAB supports multiple home visitation programs Meetings facilitate referrals process and systems coordination	Strong leadership and funder participation Limited team involvement and cross-sector representation	Single CAB supports multiple NFP programs in the same community Diverse representation from across sectors	Strong healthcare representation Limited community engagement

### Electronic supplementary material

Below is the link to the electronic supplementary material.

Supplementary Material 1

## Data Availability

The data collected and analyzed during the current study are not publicly available due to confidentiality and privacy of our participants, but de-identified data are available from the corresponding author on reasonable request.

## Abbreviations

CAB: Community Advisory Board
EMR: Electronic medical record
LHD: Local health department
MOU: Memorandum of Understanding
NFP: Nurse-Family Partnership
NHV: Nurse home visitor
US: United States
WIC: Supplemental Nutrition Program for Women, Infants, and Children
