# Supplementary material for: Structural and relational factors for successful cross-sector collaboration in home visiting: a multiple case study
Source: BMC Health Serv Res. 2024 Mar 8;24:316. doi: 10.1186/s12913-024-10719-4 (PMC10921672; doi:10.1186/s12913-024-10719-4)
Supplement: Supplementary file 1 — Supplementary Material 1 [file 12913_2024_10719_MOESM1_ESM.docx]

**Appendix A**. Table of study participants by role and case

| **Participant Role** | ***N*** | **Participant details** | **% of total study sample** |
| --- | --- | --- | --- |
| *Site 1* | 19 |  | 24% |
| Nurse-Family Partnership | 9 |  | 11% |
| Nurse home visitor, (*n*) | 6 | N/A |  |
| Nurse supervisor, (*n*) | 1 | N/A |  |
| Administrator, (*n*) | 1 | Health department chief nursing officer |  |
| Nurse consultant, (*n*) | 1 | Supports statewide program implementation |  |
| Collaborative Providers | 7 |  | 9% |
| Public health, (*n*) | 2 | WIC^a^ director and Health department public health services supervisor |  |
| Medical care, (*n*) | 2 | Care management supervisor and RN case manager |  |
| Social services, (*n*) | 3 | Outreach librarian, program manager, and family support coordinator |  |
| Clients | 3 |  | 4% |
| Current client, (*n*) | 1 | N/A |  |
| Graduated client, (*n*) | 2 | One is Spanish-speaking |  |
| *Site 2* | 14 |  | 18% |
| Nurse-Family Partnership | 9 |  | 11% |
| Nurse home visitor, (*n*) | 6 | N/A |  |
| Nurse supervisor, (*n*) | 1 | N/A |  |
| Administrator, (*n*) | 1 | N/A |  |
| Nurse consultant, (*n*) | 1 | Supports program implementation |  |
| Collaborative Providers | 2 |  | 3% |
| Medical care, (*n*) | 2 | Patient navigator and obstetrics nurse manager |  |
| Clients | 3 |  | 4% |
| Current client, (*n*) | 2 | N/A |  |
| Graduated client, (*n*) | 1 | Recently completed the program |  |
| *Site 3* | 14 |  | 18% |
| Nurse-Family Partnership | 8 |  | 10% |
| Nurse home visitor, (*n*) | 5 | N/A |  |
| Nurse supervisor, (*n*) | 1 | N/A |  |
| Administrator, (*n*) | 1 | Oversees maternal child health division |  |
| Program staff, (*n*) | 1 | Records technician |  |
| Collaborative Providers | 3 |  | 4% |
| Public health, (*n*) | 1 | WIC supervisor |  |
| Social services, (*n*) | 2 | Children’s council funding agency |  |
| Clients | 3 |  | 4% |
| Current client, (*n*) | 3 | N/A |  |
| *Site 4* | 17 |  | 21% |
| Nurse-Family Partnership | 7 |  | 8.5% |
| Nurse home visitor, (*n*) | 3 | N/A |  |
| Nurse supervisor, (*n*) | 1 | N/A |  |
| Administrator, (*n*) | 1 | N/A |  |
| Organizational staff, (*n*) | 2 | Senior leadership and Intake coordinator |  |
| Collaborative Providers | 7 |  | 8.5% |
| Public health, (*n*) | 2 | WIC Director and state health representative |  |
| Medical care, (*n*) | 3 | Hospital social worker and clinical psychologist |  |
| Social services, (*n*) | 2 | Child welfare specialist and early intervention director |  |
| Clients | 3 |  | 4% |
| Current client, (*n*) | 1 | N/A |  |
| Graduated client, (*n*) | 2 | Both are Spanish-speaking |  |
| *Site 5* | 15 |  | 19% |
| Nurse-Family Partnership | 9 |  | 11% |
| Nurse home visitor, (*n*) | 5 | Two from Team 1, three from Team 2 |  |
| Nurse supervisor, (*n*) | 2 | One per team |  |
| Administrator, (*n*) | 1 | Oversees other home visiting programs and substance use treatment program |  |
| Program staff, (*n*) | 1 | Administrative assistant |  |
| Collaborative Providers | 3 |  | 4% |
| Public health, (*n*) | 1 | Medicaid home visiting supervisor |  |
| Medical care, (*n*) | 2 | Clinic nurse practitioner and substance use treatment program supervisor |  |
| Clients | 3 |  | 4% |
| Current client, (*n*) | 3 | N/A |  |

^a^Supplemental nutrition program for Women, Infants and Children

**Appendix B**. Thematic interview guide

| **Theme** | **Subtopics** | **Sample Probes** |
| --- | --- | --- |
| Introduction | Professional background  Current role and responsibilities  Organizational details and functions | What is the organizational structure like? How many people work here?  How long has Nurse-Family Partnership (NFP) been implemented by your organization?  How would you describe your organization in terms of being a local health department, community based organization, or health system/hospital? Does your organization provide healthcare services; what types of services?  What other programs are offered through your organization, such as nutrition, other home visiting or maternal child health services? How is eligibility for these various services determined? |
|  | Community and population served | Tell me a little bit about the community your site serves. What are some general characteristics of the population your site serves? |
|  | Community Advisory Board | Tell me about your site’s Community Advisory Board (CAB) and how it supports NFP implementation in your community. Who sits on the board (i.e. family voice)? How is family voice incorporated? How does *your* CAB support NFP implementation? How effective is it? What would make it more effective? What does a typical CAB meeting involve? |
| Integration with {Provider type identified from 2020 survey} | Shared physical space and personnel | To what extent do you share physical space with {provider type}? What about sharing of personnel or sharing of other resources? Do you have badge access to the {clinic/location} accessed by your clients? |
|  | Shared information | How do you share information with {provider type}? Do you have a shared electronic record system? Are you able to receive NFP referrals through the shared system ? If so, is this effective? How, so?  Do you call, email or fax care plans? How does this process work? Do you have a feedback loop about client needs and progress between both organizations? How is this completed?  Do nurses obtain a release of information? |
|  | Policies | Do you have policies or written/formal agreements to interact with {provider type} such as for making or receiving referrals, consenting to share information, coordinating care, etc.? [If they have one, ask for a copy] |
|  | Financial Incentives | What payment mechanisms exist between your organization and {provider type}? Do they fund NFP implementation? Do they reimburse for home visits? |
| Coordination with {Provider type identified from 2020 survey} | Outreach | Tell me about your outreach with {provider type}. Are nurses assigned to outreach to specific {provider type}? How often is this done? What’s working? What’s not? |
|  | Referrals process | Tell me about how you receive referrals for NFP from {provider type} (including characteristics of clients referred). Are risk factors identified? How are clients prioritized?  Tell me about how you refer clients to {provider type}. What is the process like? What works? What doesn’t?  (*If have support staff*) How do the support staff participate in NFP referrals from and referrals to {provider type}? |
|  | Knowledge and Attitudes | Tell me about the knowledge and awareness {provider type} have of NFP. How can this be improved? What knowledge and awareness of {provider type} does your organization have? What would you like to know more about? |
|  | Values and Trust | Do you believe that {provider type} and NFP share values in addressing client needs? To what extent do you trust each other to address client needs? |
|  | Mission Alignment | Do you believe that {provider type} and your organization share the same mission and goals? If so, how? |
|  | Leadership and Champions | To what extent do the leaders in your organization support your collaboration with {provider type}? Tell me how your organization’s leadership perceives collaboration with {provider type}. Is collaboration with them important? Why or why not?  Are there champions of NFP among the {provider type} in your community? What do they do to support NFP? Why do they support NFP? |
|  | Care Coordination | Tell me about care coordination in your site with {provider type}. Describe the communication between you and {provider type} to address client needs. How do you interact? Is there a specific contact person you work with? Do the nurses know who to contact? Who from the {provider type} actually communicates with you? Do you get a release of information? How does the {provider type} know they have someone in NFP and how they can contact NFP?  Do you do case conferencing or joint visits? What does that look like? How do you determine roles/responsibilities in caring for your clients’ needs? How are decisions made? |
| Successes | Examples of successes | Tell me about any examples of major successes your site has had in collaborating with community providers. Are there champions of NFP among the {provider type} in your community? What do they do to support NFP? Why do they support NFP? |
|  | Examples of care coordination | Tell me about any examples of successful care coordination in your site with {provider type}*.*  Tell me about any examples of successful care coordination in your site with other community providers. |
| Challenges | Challenges in collaboration | Tell me about any examples of challenges your site has had in collaborating with community providers. |
|  | Other relevant topics | Is there competition with other programs? Is there anything else you want to share with us about collaborating with other community providers? Do you have any questions for me? |

**Appendix C**. Details of each case

**Case 1: Rural health department in Western US**

*Context.* This Nurse-Family Partnership (NFP) site was selected for participation as an exemplary case due to their reported strong integration and coordination with women’s care providers based on the 2020 survey. In this site, the NFP program has been implemented by the local health department (LHD) since 2001 under the Clinical and Community Health Division, alongside a program for children with special healthcare needs and a family planning clinic. The NFP team is composed of the nurse supervisor, an administrator, and six nurse home visitors (NHV). The team is funded by the state to serve 150 clients. The LHD’s family planning clinic, alongside local federally qualified health centers (FQHCs), the university health system, and behavioral health centers offer the majority of medical care services for NFP clients and their children.

*Community and population served.* This site serves the third largest county in the state with an urban center and primarily rural/agricultural surrounding areas. The county has the highest incidence of child maltreatment in the state. The NFP team visits clients who are White or Hispanic/Latin-X, as well as refugee populations from Somalia and Burma. Other client characteristics include lower income, adolescents, substance use disorder and misuse, mental illness, and low educational attainment.

*Integration with women’s care.* This community has a regional health alliance that includes the LHD, local FQHCs, and the local behavioral health center. The alliance shares an electronic medical record (EMR) system that allows providers to communicate with one another by “flagging” patient charts for scheduling appointments and/or requesting follow-up communications. Providers’ ability to exchange referrals through the EMR allows for efficient exchange of referrals for clients who need to receive care from any of the entities and services that are part of the alliance. NFP is also located in the same building as the family planning clinic located at the LHD. This co-location and close proximity mean NHVs can access the clinic via their keycard and walk into clinic rooms to see a client when needed. The NFP program is less integrated with women’s care providers from the university health system and a different FQHC system, where a release of information is necessary for NHVs to communicate with their providers about mutual clients.

*Coordination with women’s care.* NHVs from this site primarily coordinate with women’s care providers from three main entities: the LHD family planning clinic, local FQHCs, and the local behavioral health center. NHVs coordinate with the family planning clinic for NFP program referrals where they can be contacted directly by clinic via phone during a client’s visit to the clinic, typically to confirm a pregnancy. With the local FQHCs, NFP share a common vision to serve low-income first-time parents. Most FQHC staff and providers are aware of NFP, though many have only a basic understanding of the purpose of the program and how they serve pregnant people and their children. There is leadership support for coordination, and the obstetrics case manager is a champion of the NFP program who regularly communicates with the NFP nurse supervisor. FQHC providers were described to be very responsive to NHV questions or concerns regarding mutual clients, especially regarding physical or mental health needs. NHVs can attend prenatal appointments with their clients, view appointments in the shared EMR, and “flag” providers in the EMR for requests or concerns. NHVs also have a direct contact person at the local behavioral health center. They coordinate with the maternal mental health specialist who participates in monthly NFP case conferences where they determine if a client’s mental health concerns are specifically related to maternal or postpartum needs, such as concerns over bonding with a newborn, or if they are general mental health needs, such as working through a traumatic experience.

*Community Advisory Board (CAB) and other collaborations.* Beyond strong coordination with women’s care providers, this NFP site has strong coordination with other program and supports including the LHD social worker for client resources, Supplemental nutrition program for Women, Infants, and Children (WIC; which is not implemented by the LHD) for clients’ nutritional needs, United Way for client items (like diapers, wipes, car seats), the local library, and a local university reading program. These relationships were highly evident based on these organizations’ eagerness to be interviewed for the case study and their engagement in the site’s CAB, which was described to be effective in meeting the needs of the NFP program. The CAB meets quarterly via video conferencing and is chaired by the NFP nurse supervisor. All CAB members stated that there is a shared commitment to provide excellent care to the population that they serve and a desire to stay connected. Serving on the CAB was described to be beneficial for all members because it helps to bridge the gap between families with needs and programs. CAB meetings help to facilitate communication between the programs regarding resources and events relevant for a common client population. The CAB invites NFP clients to participate in the final meeting of the year which boosts morale, motivates members, and reminds them of “why they do the work they do”.

**Case 2: Urban health system in South Central US**

*Context*. This NFP site was selected for participation as an exemplary case due to their reported strong integration with women’s care and pediatric care providers. In this site, the NFP program has been implemented by a teaching health system since 2008, under the women’s services line along with obstetrics and gynecology, the Centering program, adolescent health, lactation, and a substance use program for mothers on methadone. The NFP team consists of the nurse supervisor, an administrator, eight NHVs, and a data entry clerk. The team is funded to serve 200 clients through the federal Medicaid block grant. The health system has 12 satellite ambulatory clinics, of which 10 offer women’s care and pediatric services where NFP clients and their children receive medical care. A medical insurance navigator is available to enroll patients, and obstetrics patient navigator welcomes patients into the system, bridging the gap between clients and resources.

*Community and population served.* This site serves the second largest city in the state in an urban-suburban core within 25-mile radius of the NFP office. The city has the highest rate of domestic violence and child maltreatment in the state. Twenty percent of NFP clients are monolingual Spanish-speaking, and 10-15% are undocumented or refugees. All clients are Medicaid-eligible or uninsured. Other client characteristics include having mental health challenges, substance use, physical health risks, and housing instability.

*Integration with women’s and pediatric care.* The NFP office is located on the medical campus, across from the women’s health facility which is the system’s largest satellite ambulatory clinic. This co-location means that NHVs have badge access to the hospital and clinics where NFP clients receive their care. Because NFP and the health system are part of the same organization, it allows for sharing of information once consent forms and release of information are signed. NHVs have read-only access to the shared EMR to view clinic notes and send messages, while the nurse supervisor has full access. NHVs use the EMR to look up patient appointments and review patient weights and visit notes from providers.

*Coordination with women’s and pediatric care.* Since NFP is part of the women’s health service line at the health system, they share goals of compassion, collaboration, and education for patients as well as shared outcomes of healthy pregnancies and deliveries. The NFP nurse supervisor is also included in management meetings within the same service line which allows for exposure of the program. The NFP program receives the majority of their referrals from the obstetrics patient navigators based within the ambulatory clinics. Beyond referrals, NHVs coordinate with patient navigators to “track down” clients who may have missed appointments. There were multiple individuals from the largest clinic who were identified as NFP champions, including the obstetrics clinic supervisor, physician assistant, and patient navigator who tell patients about the program, submit referrals, and help to coordinate care. Many NHVs have personal relationships with the clinic front-line staff which facilitate communications, and they are able to support clients to navigate the health system. At the same time, the NFP nurse supervisor and the clinic supervisor coordinate regularly via phone and email. In general, there is less coordination with pediatric care providers. Interactions with pediatric care providers centers around scheduling well-child visits or referrals to early intervention. The pediatric patient navigators are situated within the hospital rather than the clinic, and no NHVs described communicating with these navigators.

*CAB.* This NFP site shares a CAB with two other NFP sites located in the same community. All three NFP sites are represented on the CAB and aligned to serve the community, which helps to minimize the number of meetings for individuals who would otherwise participate in independent CABs for each NFP site. CAB members represent medical care, social work, education, social services, Medicaid, juvenile detention, and early childcare. The three NFP sites each see value in sharing a CAB because they can compare and contrast measures between sites to address why one site may be having a problem with a particular performance measure. Other successes include the use of virtual meetings which has increased member participation and formal written bylaws and goals that helped form a legislative strategic plan to sustain the NFP program.

**Case 3: Urban health department in Southeastern US**

*Context*. This NFP site was selected for participation as an exemplary case due to their reported strong coordination with WIC and child welfare and integration with women’s care providers. The NFP program at this site has been implemented since 2012 by the LHD under the Maternal Child Health (MCH) Division, alongside four other home visiting programs, a family counseling service, and WIC.  The NFP team is composed of the nurse supervisor, an administrator, six NHVs, and a records technician and supported by a contracted mental health counselor who conducts joint visits for in-home counseling. The team is funded by  a non-profit children’s council funding agency (a legislative-required property tax funded organization focused on child maltreatment prevention and early childhood development) to serve 120 clients. The LHD has six health centers that offer pregnancy confirmation services, with one center that offers obstetric care services for pregnant people. Outside of the LHD, there are two major birthing hospitals and local FQHCs where NFP clients and their children receive medical care.

*Community and population served.* This site serves the third largest county in the state with urban and suburban areas. The county has the highest incidence of child maltreatment in the state. The NFP team visits clients who are White, Black, or Hispanic/Latin-X. Other client characteristics include being undocumented, adolescents, having previous adverse childhood experiences, having high mental health needs, substance use and misuse (cigarette use and legal medical marijuana use), lack of housing, lack of social support, intimate partner violence, and physical health risks.

*Integration and coordination with women’s care.* The LHD has a shared electronic medical record (EMR) system that allows all providers within the department (including NFP, health centers, and WIC) to see if a visit was made. Visit notes must be requested from the provider, and clients sign a release of information at enrollment for NHVs to request these records. NHVs also have badge access to the health centers. NHVs and women’s care providers share goals to care for pregnant people, deliver healthy babies, and connect families to needed resources.

            In addition to integration, we found that NHVs in this site had strong coordination with women’s care providers. Many NHVs had personal relationships with the local women’s care providers which facilitated program referrals and care coordination. The LHD health center that offers obstetrics services will reach out to the NFP nurse supervisor directly if a client is interested in enrolling. Because the NFP supervisor previously worked at the health center, NHVs will ask her for support to connect with providers for communications, typically to set clients up with Medicaid or communicate about patients who consistently miss appointments. One of the NHVs also has personal relationships with health center staff and a long history with the LHD; she is often asked  by women’s care providers to help reach patients, schedule appointments, and refer to housing resources. She communicates primarily with the women’s care providers via encrypted emails rather than through the EMR system. If she marks an email as “urgent”, she typically receives a quick response.

*Coordination with child welfare.* Although the survey results indicated strong NFP coordination with child welfare in this site, our interviews identified collaboration with child welfare to fall into two major areas: shared mission and data integration. Child welfare was privatized in this community, where child protective investigations were conducted by the local sheriff office. Both NFP and child welfare value safe pregnancies, child health and safety (i.e. appropriate home environment), and for families to succeed. LDH leadership felt that NFP and child welfare share the common goals of primary (preventing families from entering the system) and tertiary prevention (if a family is in the system, to connect them with wraparound services). The NFP data technician has access to the child welfare database where she is able to run reports and see if a report was opened or closed and see information on the investigation. NFP clients are asked to provide consent at enrollment for NFP to be able to look at their previous engagement with child welfare if applicable.

            The NFP program at this site has additional relationships with a related but separate entity focused on child maltreatment prevention. This agency provides funding for NFP to be implemented, supports NFP implementation and consultation, and champions the program in the community.

*Coordination with WIC.* In this site, NHVs primarily coordinate with WIC through referrals. All NHVs provide clients with a pamphlet on WIC services and phone numbers to contact WIC through the general hub phone line. Clients then enroll on their own as they are empowered to advocate for themselves and WIC has a streamlined enrollment process. NHVs respect WIC providers for their expertise in breastfeeding and nutrition and have shared goals to support healthy mothers and babies. Despite co-location and close proximity, there is little coordination of care beyond referrals to WIC.

**Case 4: Community-based organization in Northeastern US**

*Context.* This NFP site was selected because of their reported high levels of coordination with substance use treatment and child welfare services. The NFP program has been implemented by a non-profit community-based organization (CBO) with a long history and presence in their community since 2009. The CBO was formed in the late 1880’s as a safe house for children experiencing abuse or neglect and has evolved to a large organization housing 40 programs with one administration building and 12 satellite sites. All programs within the CBO use an internal EMR system for communication and client data and use a central intake system. The CBO has a strong presence in their community and are well-known and respected among healthcare and community service providers throughout the state. NFP is organized within the home visitation division of the organization which also houses Early Head Start, Head Start, childcare sites, foster care, adoption, family preservation services, and supports for families experiencing substance use. The NFP team is made up of a nurse manager, nurse supervisor and six NHVs, including two who are bilingual in Spanish and Spanish, Creole and Portuguese respectively. The NFP program is funded to serve 200 clients through the federal Maternal, Infant, and Early Childhood Home Visiting Program under the jurisdiction of the state health department, who administers the financial contracts, oversees quality improvement, monitors program outcomes, coordinates training and education for all NHVs, and leads the shared CAB for all home visitation agencies in the state.

*Community and population serviced*. This site serves urban and suburban areas, though some families live in rural areas. Because the state is small, they function much like a small town or community in terms of the presence of community leaders, healthcare, and social services providers where most major players are aware of one another and can rely on each other to ensure the well-being of the populations that they serve. The NFP population is predominantly low-income, experiencing homelessness, have low educational attainment, and/or have immigrant, undocumented or refugee status from Liberia, Haiti, Nicaragua, El Salvador, and other central American countries. Many clients live in multiple family households and have difficulties enrolling in cash or food assistance programs because of their immigration status. . Most babies are born at the Women and Infants hospital, which primarily serves families on Medicaid. Social work staff within the hospital are proponents of NFP and regularly refer clients to the program.

*Coordination with substance use programs:* When asked about coordination with substance use treatment, NFP staff discussed their collaboration with a specific home visitation and services referral program for expectant parents (the “Project”) that are affected by substance use and are involved with child welfare. The Project is housed in the same organization as NFP and falls under the jurisdiction of the state health department because of their funding stream, like NFP. Typically, families are referred to the Project by child welfare, with the goal of keeping families together and linking families to services, such as counseling and medication for opioid use disorder. Because child welfare is housed within the state health department and NFP and the Project are under the jurisdiction of the state health department, they have a streamlined process of referrals and communication. State health department and child welfare leadership recognize that both NFP and the Project are part of wrap-around care for families affected by substances. Since both programs are housed within the same organization, program leadership encourages communication and collaboration; both programs easily share client information, conduct joint visits with clients, reinforce messaging to clients, and conduct case conferences to discuss specific client needs. The Project staff conduct home visits with families after their baby is born to ensure they have the right supports in place, including referring to NFP. Their staff are also BSN trained nurses, are trained in substance use and child welfare risks assessment and monitoring, provide support to prevent relapse, and aftercare services post-child welfare involvement. Because the NFP NHVs do not receive training related to substance use, they can call on the Project staff regarding concerns about substance use or relapse in their clients. NFP clients are usually already enrolled in the Project and can communicate with Project staff to learn about the client’s needs as it relates to substance use.

*Coordination with child welfare.* According to child welfare, their mission is to keep families together and have less of a punitive approach than maybe other child welfare agencies may have; they know that these families need reliable supports in place to help them succeed, much like the strengths-based approaches of NFP. Because of their connection to the state health department, child welfare knows that NFP is a reliable and respectable program for families who are involved in the child welfare system. Child welfare case workers and NFP NHVs communicate regularly about client needs or conduct joint visits on an as-needed basis. Child welfare staff know that families may feel overwhelmed by all of the requirements of child welfare engagement, so NHVs help to ease the burden on families by acting as a liaison to child welfare on their behalf. Families will sign a release of information so that NHVs can communicate with their caseworker on their progress. The NHV will usually call the social worker when clients enroll in the program to discuss the client's service plan requirements. The NHV and case worker work together to reinforce messaging, ensure consistency in messaging, and communicate with each other if there are concerns or if one cannot get in touch with the client. NHVs shared that clients appreciate their NHV being present at visits or on calls with child welfare, because the NHV will help clients advocate for themselves and demonstrate that they are making progress on their service plan goals.

**Case 5: Urban health system in Mid-Atlantic US**

*Context*. This NFP site was selected for participation as an exemplary case due to their reported strong coordination with substance use providers and their integration with women’s care and pediatric care providers. The NFP program has been implemented by a teaching health system since 2001 under the women’s and pediatric services line along with obstetrics and gynecology, pediatrics, the Centering program, family planning, a Medicaid home visitation program, and a substance use program for mothers affected by substance use. The NFP team is composed of two nurse supervisors, an administrator, twelve NHVs, and an administrative assistant. The team is funded to serve 290 clients, with blended funding from the state’s department of Child Development and Early Learning, an Opioid Use Disorder/Substance Use Disorder (OUD/SUD) grant, and expansion dollars from the state human services department.

*Community and population served.* This site serves the sixth largest county in the state with urban areas, suburban areas, and rural/agricultural surrounding areas. The NFP team visits clients who are White, Black, or Hispanic/Latin-X as well as refugee populations from Nepal, Burma, Congo, Afghanistan, and Africa. Other client characteristics include being undocumented, adolescents, having high mental health needs, substance use and misuse (opioid and methamphetamine use), lack of housing, lack of social support, intimate partner violence, and physical health risks. Most NFP clients receive their healthcare from local FQHCs and deliver at the hospital that is a part of the health system that implements the program.

*Integration and coordination with women’s and pediatric care.* The NFP office is physically located a half block away from the medical campus, and the NHVs have badge access to the hospital. NFP and the health system being part of the same organization allows for sharing of information once consent forms and releases of information are signed. The health system has a shared EMR system that allows all providers within the department (including NFP and other home visiting programs) to see if a visit was made. NHVs chart in and have full access to the shared EMR with the health system and other home visiting programs for referrals and to view clinic notes and send messages within the system.

In addition to integration, we found that NHVs in this site had strong coordination with women’s care providers both within and outside the health system. Many NHVs had personal relationships with the health system’s women’s care providers and other home visiting programs which facilitate program referrals and care coordination. In addition to the health system providers, there are seven FQHCs in the county which have providers who have full privileges to deliver babies at their hospital and full access to the shared EMR. One of the providers from the FQHC has long-standing personal relationships with several of the NFP NHVs and freely offers her cell phone number to communicate with her. NFP further shares goals with women’s care providers within and outside the health system in promoting healthy pregnancies and babies. However, NFP and women’s care providers approach care differently, where NFP focuses on client goals while women’s care often focuses on clinical standards.

Within the health system, all pregnant people are screened for eligibility for home visiting services at their first prenatal appointment. The Medicaid home visitation program at the health system receives these referrals and outreaches to the referrals, conducts a full assessment including mental health needs, and then refers them to either the NFP program, the substance use program or retain them in the Medicaid home visiting program. These referrals account for 85% of the referrals that NFP receives. If the person enrolls in NFP, the social worker from the Medicaid home visiting program continues to work alongside the NFP NHV to coordinate care. The NFP administrator also oversees the three home visitation programs within the health system which allows for greater cohesion where the programs are in constant communication.

In general, there is less coordination with pediatric care providers. Although NFP and pediatric care providers share the same goal of ensuring the health and wellbeing of the baby, most pediatricians in this community lack knowledge and awareness of the NFP program and the supports that NHVs can offer to families.

*Coordination with substance use treatment providers.* Coordination with substance use providers refers to coordination regarding clients who are dually enrolled in both NFP and the substance use home visitation program offered through their health system.  The NHVs share the same administrator with the substance use program and are co-located within the same office space.  Both programs share the same EMR and can send referrals, communicate, and view notes through the system. Despite the opportunity for care coordination, most clients are not dually enrolled in both programs. NFP and the substance use program share the same goal in supporting mothers to be the best parent. The substance use program offers mothers community support through engagement with others with similar lived experiences, and NFP provides individualized support to address family needs.
